# Cost-effectiveness of community-based artificial intelligence screening for retinopathy of prematurity

**DOI:** 10.7189/jogh.16.04357

**Published:** 2026-10-02

**Authors:** Jiayu Xu, Zhihan Zhang, Guanran Zhang, Yanlin Qu, Zhenyu Wu, Xiaodong Sun, Da He, Huixun Jia

**Affiliations:** 1 School of Public Health, Shanghai Jiao Tong University School of Medicine, Shanghai, China; 2 School of Basic Medicine, Tongji Medical College, Huazhong University of Science and Technology, Wuhan, China; 3 Department of Ophthalmology, Shanghai General Hospital, Shanghai Jiao Tong University School of Medicine, Shanghai, China; 4 School of Public Health, Fudan University, Shanghai, China; 5 Shanghai Health Development Research Center, Shanghai, China

**Keywords:** retinopathy of prematurity, artificial intelligence, telemedicine, cost-effectiveness, community-based screening, quality-adjusted life-years

## Abstract

**Background:**

Retinopathy of prematurity (ROP) is one of the leading causes of childhood blindness globally, but particularly in middle-income countries, where neonatal survival has improved faster than access to specialist ophthalmic care. Although telemedicine and artificial intelligence (AI)-assisted screening show promise for improving access to ROP screening, their economic value within decentralised health systems remains uncertain. To explore this issue, we evaluated the cost-effectiveness of community-based AI-assisted ROP screening in China compared with telemedicine and traditional bedside screening.

**Methods:**

We developed a decision tree model to estimate lifetime health outcomes and societal costs for a hypothetical cohort of 100,000 preterm infants eligible for ROP screening. We compared three strategies: community-based AI-assisted screening, community-based telemedicine screening, and bedside screening at specialist healthcare facilities. Separate parameter sets were applied for urban and rural settings. Costs were assessed from a societal perspective.

**Results:**

Community-based AI-assisted screening was the most cost-effective strategy in both urban and rural settings. In the base-case analysis, telemedicine screening dominated bedside screening (meaning it was less costly and more effective), and AI-assisted screening further dominated telemedicine screening. AI-assisted screening incurred the lowest lifetime costs (USD 1,507 per infant in urban and USD 2,581 in rural areas) and the greatest health benefits (29.7138 quality-adjusted life-years (QALYs) in urban and 29.5835 QALYs in rural areas). Compared with bedside screening, AI-assisted screening was dominant, with incremental cost-effectiveness ratios of USD −7,307 per QALY gained in urban and USD −3,688 per QALY gained in rural settings. Findings were robust across scenario and sensitivity analyses, with AI-assisted screening ceasing to be cost-effective only at extremely low treatment-requiring ROP incidence (<0.7%) or low screening coverage (11%) in rural areas.

**Conclusions:**

Community-based AI-assisted ROP screening is likely the most cost-effective strategy in China. By expanding access to early eye care while reducing societal costs, it may support equitable service delivery and offer a scalable model for other regions facing similar resource constraints.

Global demographic shifts are reshaping priorities in neonatal healthcare. With fertility rates declining and projected to remain below replacement level through 2100, more than half of countries now report total fertility rates below 2.1 [1]. This transition has shifted the focus of neonatal care from reducing mortality toward ensuring high quality survivorship [2]. Concurrently, rising maternal age and higher rates of multiple pregnancies have increased the risk of adverse neonatal outcomes [3]. Over the past three decades, global blindness due to neonatal disorders has risen by 33.5%, with retinopathy of prematurity (ROP) being the leading cause of vision loss due to neonatal disorders [4]. Early screening is, therefore, essential for preserving vision and improving long-term quality of life in preterm infants. Despite its importance, access to ROP screening remains highly uneven worldwide. Approximately 15% of countries lack universal ROP screening programmes, and 26% lack standardised screening guidelines [5]. These gaps are particularly pronounced in middle-income countries, where neonatal survival has improved faster than the availability of specialised ophthalmic services [6]. Bedside binocular indirect ophthalmoscopy, typically performed in large central hospitals, remains the most widely accepted method for ROP screening [7]. However, this approach requires substantial expertise for accurate diagnosis and staging, restricting its implementation in many healthcare facilities that otherwise provide routine neonatal healthcare [8].

Innovations in digital health offer promising alternatives. Telemedicine platforms supported by 5G networks enable real-time transmission of retinal images, overcoming geographic barriers. Furthermore, artificial intelligence (AI) deep learning models have demonstrated high sensitivity (0.71–1.00 for the proportion of actual ROP cases correctly identified) and specificity (0.74–0.99 for the proportion of non-ROP cases correctly ruled out) in ROP diagnosis, reducing reliance on scarce specialists and improving screening efficiency [9,10]. However, reported diagnostic performance varies across studies, likely due to differences in image quality, AI algorithms, and study settings. More recently, some middle-income countries have begun exploring a community-based ROP screening strategy, in which neonatal intensive care units (NICUs) without onsite ophthalmologists are equipped with retinal imaging devices and digital platforms for remote interpretation [11-13]. This approach decentralises image acquisition while centralising diagnostic expertise, extending screening to underserved and remote regions.

Despite the growing promise of telemedicine and AI, important economic evidence gaps impede widespread adoption. First, although studies from high-income countries have shown that telemedicine and AI-assisted screening are cost-effective, these analyses have primarily focused on improvements in diagnostic accuracy and workflow efficiency [14]. In contrast, in many middle-income countries, the principal value of these technologies lies not only in improving diagnostic performance, but in enabling community-level healthcare institutions to initiate ROP screening. Second, most existing economic evaluations have failed to account for nonmedical costs, including transportation, accommodation, and productivity losses associated with caregiving, potentially underestimating the true societal burden of ROP management [15].

In this study, we developed a decision tree model tailored to China’s urban–rural healthcare structure to evaluate the cost-effectiveness of a community-based ROP screening strategy. By incorporating both medical and non-medical costs, we estimated comprehensive incremental cost-effectiveness ratios (ICERs) for a cohort of 100,000 infants requiring ROP screening over a 79-year lifetime horizon. Our findings could inform evidence-based ROP screening policies in China and provide policy-relevant insights for other regions with established neonatal care capacity but limited access to ophthalmic specialists.

## METHODS

We developed a decision tree model to simulate lifetime health outcomes and economic burdens associated with ROP screening (Figure S1 in the **Online Supplementary Document**). We report our findings per the CHEERS guidelines for economic evaluation studies (Table S8 in the **Online Supplementary Document**) [16]. We performed all analyses in TreeAge Pro 2022 (TreeAge Software, Williamstown, Massachusetts, USA).

The model simulated a hypothetical cohort of 100,000 infants requiring ROP screening (gestational age <32 weeks and/or birth weight <2,000 g [17]), representing individuals generated within the decision tree model using epidemiological estimates. This cohort size has been commonly used in economic evaluations of infant screening programmes [18-20]. We classified participants as having no ROP, non-treatment-requiring ROP, or treatment-requiring ROP based on epidemiological estimates. Those without treatment-requiring ROP were assigned the ‘no visual impairment’ outcome, while those with treatment-requiring ROP received the ‘timely treatment’ (defined as diagnosis and treatment within 72 hours), ‘delayed treatment’, or ‘blindness if untreated’ outcomes, depending on screening detection and referral completion. Disease classification followed the International Classification of Retinopathy of Prematurity [21]. We defined treatment-requiring ROP as type 1 or threshold ROP requiring laser photocoagulation or intravitreal anti-vascular endothelial growth factor injection within 72 hours [22]. Infants progressing to stage 4 or 5 ROP required vitrectomy and/or scleral buckling surgery. We incorporated inputs from three paediatric retinal specialists with more than ten years of experience in ROP diagnosis and management to ensure that model assumptions and parameterisation reflected the real-world healthcare settings in China.

### Model structure

We defined three alternative ROP screening strategies based on current clinical practice and specialist input in China, which represent the assumed clinical pathways in the model for a hypothetical cohort (**Figure 1**). Community-based AI-assisted screening (AI screening) is implemented in community-based healthcare facilities, including primary- and secondary-level hospitals in China. In this strategy, trained ophthalmic technicians use retinal imaging systems to capture standardised fundus images [23]. Images are transmitted to a regional reading centre, where AI models perform initial image analyses, followed by independent review by two retinal specialists, who provide referral recommendations within 24 hours. Community-based telemedicine screening (telescreening) is also conducted in community-based healthcare facilities. Fundus images are captured and transmitted using the same approach as AI screening. Two retinal specialists independently review the images and provide referral recommendations within 24 hours, without AI pre-screening. Since the imaging devices and acquisition protocols are the same as those used in AI screening, differences in screening performance between the two community-based strategies reflect the presence or absence of AI pre-screening. Bedside screening is performed in specialist-based healthcare facilities, corresponding to tertiary hospitals in China. Retinal examinations are conducted on site by paediatric retina specialists using binocular indirect ophthalmoscopy or retinal imaging systems to directly assess retinal conditions [17].

**Figure 1:**
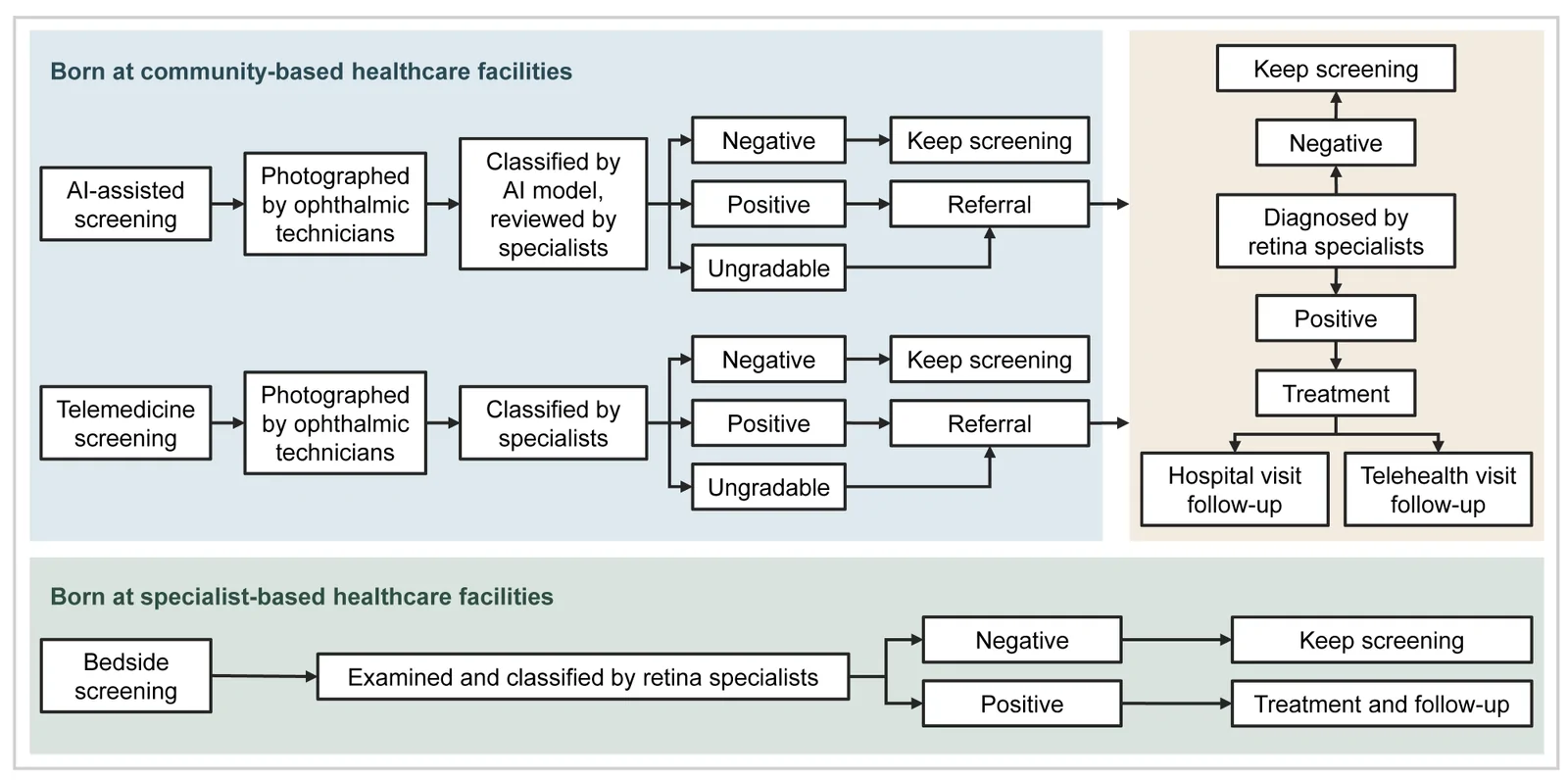
Eye care workflows for screening, diagnosis, treatment, and follow-up across three retinopathy of prematurity screening strategies. AI – artificial intelligence.

Under the community-based screening strategies, infants with positive results or ungradable retinal images should be referred to specialist-based facilities for further diagnosis and potential intervention. All screened infants are assumed to undergo an average of four screening examinations before discharge, based on empirical data from clinical practice [24].

### Model parameters

We obtained event probabilities and health-related quality-of-life values through a literature review, and derived cost inputs from Chinese medical fee schedules, published literature, and a nationwide survey (Method S1 in the **Online Supplementary Document**). To reflect regional disparities in economic development and healthcare access, we specified separate parameter values for urban and rural settings.

#### Event probability

Population-based estimates of the incidence of treatment-requiring ROP in China remain sparse, with few published studies reporting on this outcome. Therefore, we employed a two-step estimation of applying the overall ROP incidence (n/N = 165/1,833, 9.0%) from a prospective screening cohort [25] and multiplying it by the proportion of treatment-requiring cases among diagnosed ROP patients (n/N = 1,330/4,770, 27.9%) [26], yielding a base-case treatment-requiring ROP incidence estimate of 2.5%. This approach assumes comparability between the source populations, as both studies are nationwide multicentre cohorts with similar inclusion criteria based on national ROP screening guidelines.

Based on published data on the national distribution of NICU beds and regional epidemiological studies, the proportion of births at specialist-based healthcare facilities was estimated at 63%, with the remaining 37% located at community-based facilities [27]. Coverage rates for bedside screening at specialist-based facilities were estimated at 74% in urban and 31% in rural settings [28,29]. However, among infants born at community-based facilities, who were not routinely included in bedside screening, opportunistic case-finding rates (*i.e.* the proportion of ROP cases detected outside a formal screening programme when infants are examined for other clinical reasons) were substantially lower, at 25% in urban and 11% in rural settings [28,29]. In contrast, under telemedicine and AI screening strategies, screening coverage for these infants was assumed to be comparable to that achieved at specialist-based facilities. Compliance with referral and diagnostic examination was estimated at 63% in urban and 21% in rural settings [30,31], while treatment compliance among diagnosed cases was 90% and 79%, respectively [31,32].

Regarding screening performance, paediatric retinal specialists were assumed to achieve a sensitivity of 0.92 and specificity of 0.99 for identifying treatment-requiring ROP [33]. AI-based classification for referral-warranted ROP (*i.e.* zone I ROP, stage 3 or worse, or plus disease) demonstrated a sensitivity of 1.00 and specificity of 0.98 [33]. The retinal image readability rate was estimated at 88% [34].

#### Health-related quality of life

We used quality-adjusted life-years (QALYs) to capture health-related quality of life, calculating them by applying utility values associated with each outcome over a life expectancy of 79 years [35], discounted at an annual rate of 3% [36]. Here, we set utility values, defined as a measure of quality of life on a scale from 0 (death) to 1 (perfect health), at 0.97 for infants with no visual impairment, 0.70 for those receiving timely treatment, and 0.27 for those who were blind [37,38]. For delayed treatment cases, utility was estimated at 0.52, corresponding to an average Snellen visual acuity of 20/1,500 [39] based on the transformation formula *utility =* 0.374 × *Snellen visual acuity* + 0.514 [40].

#### Costs

We adopted a societal perspective for cost estimation. During the screening phase, only direct medical costs were included, as screening was primarily conducted during hospitalisation. In subsequent phases, additional expenses were incorporated, including food, transportation, and accommodation, as well as productivity losses for accompanying parents or caregivers. Thus, both direct nonmedical costs and indirect costs were considered. For infants progressing to blindness, the economic burden was estimated at USD 8920 in the first year and USD 3604 in subsequent years, discounted annually at 3% [29].

We recorded all costs in CNY and converted them to USD using the 2024 annual average exchange rate (USD 1 = CNY 7.1217) [41] (**Table 1**; Tables S3–S6 in the **Online Supplementary Document**).

**Table 1:** Costs of retinopathy of prematurity screening, diagnosis, treatment, and follow-up

	Urban settings			Rural settings		
	**AI screening**	**Telescreening**	**Bedside screening**	**AI screening**	**Telescreening**	**Bedside screening**
**Screening costs in USD**	257	281	236	250	268	227
**Diagnosis costs in USD**	348	348	0	786	786	0
**Treatment costs in USD**						
Timely treatment	759	759	759	720	720	720
Delayed treatment	3,481	3,481	3,481	3,313	3,313	3,313
Blindness	8,920	8,920	8,920	8,920	8,920	8,920
**Follow-up costs in USD**						
Timely treatment	539	539	616	588	588	665
Delayed treatment	1,001	1,001	1,144	1,092	1,092	1,235
Blindness	3,604	3,604	3,604	3,604	3,604	3,604

AI – artificial intelligence

### Cost-effectiveness analysis

We compared lifetime costs and health outcomes across screening strategies. Then, we calculated ICERs as the difference in costs divided by the difference in QALYs between strategies. Cost-effectiveness was evaluated against willingness-to-pay (WTP) thresholds of USD 14,033 for urban settings and USD 12,251 for rural settings, derived from China’s *per capita* gross domestic product (USD 13,445) with adjustments for the urbanisation rate (67%) and the urban-rural income ratio (1:0.43) (Method S2 in the **Online Supplementary Document**) [41]. Following World Health Organization recommendations and China-specific economic evaluations, we considered a strategy to be cost-effective if the ICER fell below the corresponding WTP threshold, and dominant if it achieved both lower costs and greater health benefits [42].

We conducted scenario analyses to explore alternative assumptions. First, we applied a shortened life expectancy of 10 years to account for substantial non-ophthalmic comorbidities that may reduce long-term survival in preterm infants. Second, to reflect regional heterogeneity in clinical practice (*i.e.* screening criteria, oxygen therapy protocols, and neonatal intensive care quality), we varied the incidence of treatment-requiring ROP from 0.2% to 14.0% [43-45]. Third, considering potential implementation delays, we varied the screening coverage of telemedicine and AI-assisted strategies from opportunistic case-finding levels to coverage comparable with specialist centres to capture uncertainty during early-stage implementation. Fourth, to address the wide range of AI diagnostic performance (sensitivity of 0.71–1.00, specificity of 0.74–0.99 [9]), we evaluated an extreme scenario in which AI screening operated autonomously without expert verification, using the lower-bound sensitivity and specificity estimates to quantify the economic consequences of AI performance variability.

Lastly, we conducted multiple sensitivity analyses to evaluate the robustness of the results. In one-way sensitivity analyses, probabilities and utilities were varied by ±10%, costs for bedside screening, diagnosis, treatment, and follow-up were varied by ±20%; and costs for telemedicine and AI screening were varied by ±50% to reflect uncertainty related to equipment and digital platform investments [46]. Probabilistic sensitivity analyses were conducted using 1,000 Monte Carlo simulations (Tables S1 and S2 in the **Online Supplementary Document**), with *β* distributions applied to probabilities and utility parameters (bounded between 0 and 1) and *γ* distributions applied to cost parameters (nonnegative and right-skewed) [46].

## RESULTS

The base-case analysis showed that telescreening dominated bedside screening and AI screening dominated telescreening, making AI screening the most cost-effective strategy for ROP management in both urban and rural settings (**Table 2**). The expected lifetime cost per infant under AI screening was USD 1,507 in urban and USD 2,581 in rural areas, corresponding to health gains of 29.7138 and 29.5835 QALYs, respectively. In a hypothetical cohort of 100,000 infants, AI screening was estimated to save USD 0.21–25.14 million, increase 14–3,440 QALYs, and prevent up to 257 cases of lifetime blindness, depending on the comparator and setting.

**Table 2:** Cost-effectiveness under base-case and scenario life expectancy assumptions

	Calculation method	Costs per person in USD	Incremental costs per 100,000 people in USD	Effectiveness per person in QALYs	Incremental effectiveness per 100,000 people in QALYs	ICERs in USD per QALY gained	Blindness per 100,000 people	Blindness avoided per 100,000 people
**Base-case analysis strategy (79-year life expectancy)**								
Urban settings								
*AI screening*		1,507		29.7138			1,063	
*Telescreening*	AI *vs.* telescreening	1,514	−722,367	29.7126	114	−6,337	1,063	0
*Bedside screening*	AI *vs.* bedside	1,758	−25,135,052	29.6794	3,440	−7,307	1,320	257
	Telescreening *vs.* bedside	1,758	−24,412,685	29.6794	3,326	−7,340	1,320	257	
Rural settings								
*AI screening*		2,581		29.5835			2,067	
*Telescreening*	AI *vs.* telescreening	2,583	−214,239	29.5833	14	−15,303	2,067	0
*Bedside screening*	AI *vs.* bedside	2,596	−1,515,603	29.5794	411	−3,688	2,097	30
	Telescreening *vs.* bedside	2,596	−1,301,364	29.5794	397	−3,278	2,097	30	
**Scenario analysis strategy (10-year life expectancy)**								
Urban settings								
*AI screening*		656		8.4253			1,063	
*Telescreening*	AI *vs.* telescreening	663	−722,367	8.4250	32	−22,574	1,063	0
*Bedside screening*	AI *vs.* bedside	701	−4,564,109	8.4155	975	−4,681	1,320	257
	Telescreening *vs.* bedside	701	−3,841,741	8.4155	943	−4,074	1,320	257	
Rural settings								
*AI screening*		926		8.3883			2,067	
*Telescreening*	AI *vs.* telescreening	928	−214,239	8.3883	4	−53,560	2,067	0
*Bedside screening*	AI *vs.* bedside	917	941,093	8.3872	117	8,044	2,097	30
	Telescreening *vs.* bedside	917	1,155,332	8.3872	113	10,224	2,097	30	

AI – artificial intelligence, ICER – incremental cost-effectiveness ratio, QALY – quality-adjusted life year

Under the scenario assuming a shortened life expectancy of 10 years, AI screening remained dominant in urban settings (ICERs ranging from −22,574 to −4,681 USD per QALY gained) and cost-effective in rural settings (ICERs ranging from −53,560 to 8,044 USD per QALY gained) compared with bedside screening and telescreening (**Table 2**). Across a broad range of treatment-requiring ROP incidence rates, AI screening ceased being cost-effective only when the incidence fell below 0.7% in rural settings (**Figure 2**). When varying community-based screening coverage, AI screening remained cost-effective across the full range in urban settings and was not cost-effective (ICER of 13,960 USD per QALY gained) only at the 11% coverage level (equivalent to the opportunistic case-finding rate) in rural settings (Figure S2 in the **Online Supplementary Document**). In the extreme scenario of autonomous AI screening with lower-bound sensitivity and specificity, both community-based strategies remained dominant over bedside screening, but AI screening was not cost-effective compared with telescreening, incurring higher costs and lower effectiveness (Table S7 in the **Online Supplementary Document**).

**Figure 2:**
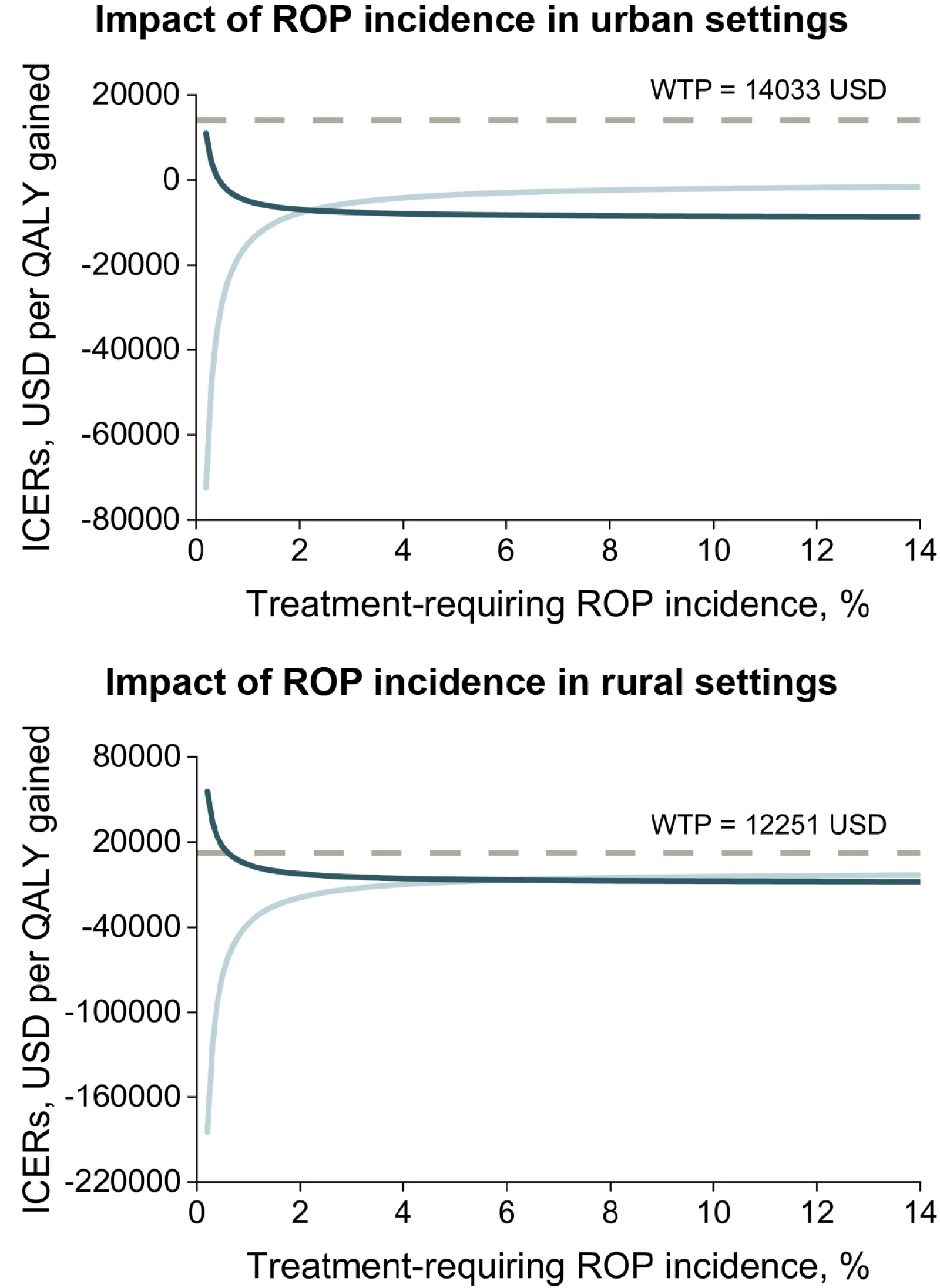
Scenario analysis of treatment-requiring retinopathy of prematurity incidence in urban and rural settings. Dark blue lines represent AI screening compared with bedside screening, while light blue lines represent AI screening compared with telemedicine screening. AI – artificial intelligence, ICER – incremental cost-effectiveness ratio, QALY – quality-adjusted life year, ROP – retinopathy of prematurity, WTP – willingness-to-pay.

Sensitivity analyses confirmed the robustness of the base-case findings. The tornado plot identified the cost of AI screening (ICER ranging from −8,330 to −6,284 USD/QALY in urban and from −7,171 to −199 in rural settings), lifetime cost of blindness (ICER ranging from −9,109 to −5,504 in urban and from −5,485 to −1,884 in rural settings), and utility associated with timely treatment (ICER ranging from −8,737 to −6,279 in urban and from −4,408 to −3,166 in rural settings) as the top three drivers of ICERs when comparing AI screening with bedside screening (**Figure 3**; Figure S3 in the **Online Supplementary Document**). The scatterplots based on 1,000 Monte Carlo simulations showed that AI screening was cost-effective in 75.7% of iterations in urban and 59.2% in rural settings (Figure S4 in the **Online Supplementary Document**). The cost-effectiveness acceptability curves further indicated that AI screening was the optimal strategy, accounting for 76.1% (urban) and 63.8% (rural) of simulations under the current WTP thresholds (Figure S5 in the **Online Supplementary Document**).

**Figure 3:**
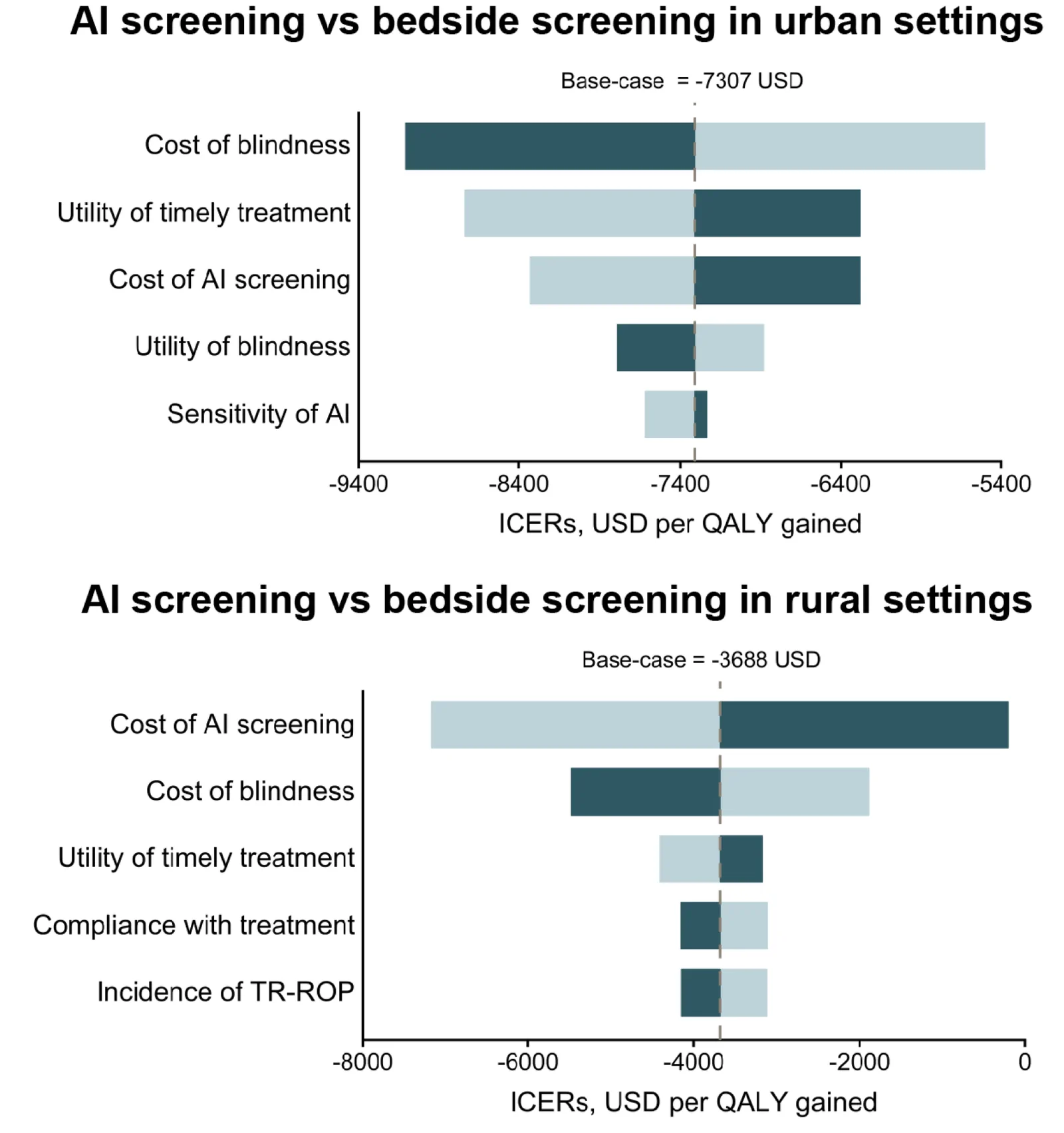
Tornado plot of one-way sensitivity analyses comparing AI screening with bedside screening in urban and rural settings. Dark blue bars represent ICERs when parameter values are set to their upper bounds, while light blue bars represent ICERs when parameter values are set to their lower bounds. AI – artificial intelligence, ICER – incremental cost-effectiveness ratio, QALY – quality-adjusted life year, TR-ROP – treatment-requiring retinopathy of prematurity.

## DISCUSSION

Our results indicate that community-based AI-assisted screening is likely the most cost-effective strategy for ROP management in both urban and rural China. Compared with telescreening and bedside screening, AI screening was associated with lower costs, greater QALY gains, and fewer cases of lifetime blindness. These findings remained robust across extensive sensitivity analyses, with AI screening ceasing to be cost-effective only when treatment-requiring ROP incidence fell below 0.7% or when screening coverage decreased to 11% in rural settings.

ROP remains a leading cause of childhood blindness worldwide [6], necessitating scalable and effective screening strategies to reduce its clinical and economic burden. Evidence from high-income countries shows that timely bedside screening and treatment can prevent up to 32 cases of lifelong blindness per 28,321 infants screened [47]. In middle-income countries, preventing a single case of ROP-related blindness is estimated to save approximately USD 195,257 in lifetime societal costs, which far exceeds the expense of ROP treatment [48]. The emergence of digital image-based screening technologies has substantially enhanced the cost-effectiveness of ROP screening. For example, a study conducted in the USA reported that telemedicine combined with AI achieved slightly lower costs (USD 15,147 *vs*. USD 15,392) and higher effectiveness (23.193 *vs*. 23.187 QALYs) than bedside ophthalmoscopy, while telescreening alone had the lowest cost (USD 15,063) with comparable effectiveness (23.192 QALYs) [14]. In contrast, our findings showed a more pronounced advantage of AI-assisted screening, with consistent dominance over both telescreening and bedside screening across urban and rural China. This difference likely reflects the limited availability and uneven distribution of paediatric retinal specialists in China, together with large screening volumes, which may amplify both the clinical and economic value of AI-assisted approaches.

This progress raises another critical question in health economics: how to make digital health-assisted screening more equitably accessible? Community-based healthcare systems represent a critical foundation for achieving equitable access. In China, community hospitals account for approximately 40% of all newborn and NICU beds nationwide [27], while community-led neonatal vaccination programmes have achieved coverage rates exceeding 95% [49]. Community-based screening programmes for adult fundus diseases have likewise demonstrated high compliance and favourable cost-effectiveness [50]. Our model evaluated a pathway in which community-level image acquisition is combined with centralised interpretation supported by AI and specialist review. This framework directly addresses the shortage of paediatric retinal specialists at the primary-care level while expanding screening accessibility.

In our analysis, we deliberately accounted for substantial urban-rural disparities by applying distinct health-related event probabilities for each setting and incorporating nonmedical costs such as transportation and productivity losses. The results consistently identified community-based AI-assisted screening as the most cost-effective strategy. This strategy works by expanding screening coverage among infants born outside specialist centres, reducing travel burden for families, and improving continuity of follow-up care. Simultaneously, it increases the efficiency of regional specialists through AI-powered pre-screening, thereby lowering economic and social barriers from both the supply and demand sides of healthcare delivery. These findings position AI screening as a viable and equitable solution for addressing the global burden of ROP, particularly in settings with limited access to ophthalmological specialists.

The generalisability of our conclusion that AI-assisted screening is the most cost-effective strategy should be approached through the adaptation of said strategies to local realities, rather than direct replication of the Chinese model. As an upper-middle-income country, China has developed advanced digital infrastructure, including widespread 5G networks, digital health platforms, and established tiered referral systems. Simultaneously, the country faces substantial within-country disparities in healthcare access and specialist availability. The consistent dominance of AI-assisted screening in rural settings suggests that this model may be applicable where community-level facilities provide the most accessible point of care and specialist resources are scarce. However, this advantage was not unconditional, as AI-assisted screening ceased to be cost-effective when the incidence of treatment-requiring ROP fell below 0.7% in rural settings. In more resource-constrained settings, simplified approaches such as portable fundus cameras and smartphone-based imaging combined with offline or semi-automated AI systems may offer feasible lower-cost alternatives [51-53]. Future studies should evaluate context-specific implementation strategies, including infrastructure requirements, workforce capacity, referral pathways, and cost structures, across settings where neonatal survival is improving but specialist eye care has not kept pace.

While community-based AI-assisted screening shows substantial promise, successful implementation will require coordinated policy support. First, policy support is needed to establish shared data platforms, define legal responsibilities, and promote equitable resource allocation [54,55]. Second, public health education should be expanded to raise awareness of neonatal eye care and build trust in digital health tools. Third, frontline healthcare workers need training in AI tools and retinal imaging devices, drawing on lessons from successful community-based screening programmes for adult fundus diseases to create standardised workflows [56]. Regional pilot programmes can further refine implementation strategies and support national scale-up.

Several limitations should be acknowledged. First, we derived key model parameters including treatment-requiring ROP incidence, image readability, and health utility values, from published studies; therefore, they may not fully reflect current clinical practice, while the cost structure of AI-based healthcare remains unstandardised. As patient data volume increases, system maintenance costs may rise, and false-positive results or ungradable images could generate additional workload, introducing uncertainty into long-term cost estimation. Nevertheless, extensive scenario and sensitivity analyses consistently showed that the base-case findings were robust across wide parameter ranges. Second, the real-world diagnostic performance of AI in ROP screening should ideally be evaluated in randomised controlled trials [57], but such evidence is currently lacking. To address this uncertainty, we modelled a conservative autonomous-AI scenario without expert verification using lower-bound sensitivity and specificity estimates. Under this assumption, AI screening remained dominant over bedside screening, but was not cost-effective compared with telescreening. This finding demonstrates the robustness of AI screening’s economic value while underscoring the practical importance of the AI pre-screening model with mandatory specialist review. Third, fundus screening in preterm infants can detect multiple ocular conditions beyond ROP, such as familial exudative vitreoretinopathy, congenital cataracts, and severe retinal haemorrhages [25]. By restricting our analysis to ROP, we may have underestimated the broader clinical value of AI screening. Future research encompassing a wider spectrum of ocular diseases would provide a more comprehensive evaluation of the value of AI-assisted neonatal eye screening programmes.

## CONCLUSIONS

Community-based AI-assisted screening represents the most cost-effective strategy for ROP management in both urban and rural regions of China. By equipping community healthcare facilities with retinal imaging systems and digital platforms, this approach improves screening efficiency, diagnostic accuracy, and equity in service delivery. Beyond supporting China’s national goals for preventing childhood blindness, this strategy may provide a valuable approach for other regions facing similar healthcare challenges in optimising resource allocation.

## Additional material


Online Supplementary Document

## Data Availability

**Data availability:** Data for this study are fully presented in the figures, tables, and Online Supplementary Document. No additional data sets were generated or analysed.
