# Supplementary material for: Cost-effectiveness of community-based artificial intelligence screening for retinopathy of prematurity
Source: J Glob Health. 2026 Oct 2;16:04357. doi: 10.7189/jogh.16.04357 (PMC13631772; doi:10.7189/jogh.16.04357)
Supplement: Supplementary file 1 [file jogh-16-04357-s001.pdf]

Supplement to: Xu J, Zhang Z, Zhang G, Q Y, Wu Z, Sun X, He D, Jia H. Cost-effectiveness of community-based artificial intelligence screening for retinopathy of prematurity. J Glob Health. 2026;16:04357.

Method S1. National survey for cost estimation

Method S2. The calculation formula of urban and rural per capita GDP

Figure S1. Decision-analytic model for retinopathy of prematurity screening strategies

Table S1. Variation ranges and probability distributions assumed for incidence, healthcare accessibility, diagnostic performance, utility values, and other model parameters

Table S2. Variation ranges and probability distributions assumed for screening, diagnosis, treatment, and follow-up costs

Table S3. Costs of retinopathy of prematurity screening in urban and rural China

Table S4. Costs of retinopathy of prematurity diagnosis in urban and rural China

Table S5. Costs of retinopathy of prematurity treatment in urban and rural China

Table S6. Costs of retinopathy of prematurity follow-up care in urban and rural China

Table S7. Cost-effectiveness in scenario analyses assuming lower AI sensitivity and specificity

Figure S2. Scenario analysis of screening coverage in community-based healthcare facilities in urban and rural settings

Figure S3. Tornado plot of one-way sensitivity analyses comparing AI-assisted screening with telemedicine screening in urban and rural settings

Table S8. Consolidated Health Economic Evaluation Reporting Standards checklist

Method S1. National survey for cost estimation

S1.1 Survey Design and Objectives

The national survey was conducted to inform the estimation of non-medical and indirect costs associated with referral, diagnostic examination, treatment, and follow-up for infants undergoing retinopathy of prematurity (ROP) screening. Specifically, the survey collected information on food and accommodation expenses, travel time, and transportation modes required to access healthcare facilities at different levels. For questionnaire acceptability and ease of understanding among respondents, all questions were framed using a general "neonatal fundus screening" scenario rather than disease-specific terminology. The full questionnaire is presented below.

Questionnaire on Access and Willingness for Neonatal Fundus Screening

Thank you for taking the time to participate in this survey.
In China, more than 10% of neonates have eye abnormalities that, if left untreated, may lead to vision loss or even blindness. Neonatal fundus screening is crucial for early detection and treatment. However, challenges persist in some regions due to unequal healthcare resource distribution and limited parental awareness of the importance of screening. This survey aims to better understand people's experiences with healthcare and their willingness to have their neonates undergo fundus screening. Your responses will help us improve these services.
The questionnaire is anonymous and will take approximately 10 minutes to complete. All responses will be kept strictly confidential and used solely for research purposes. We sincerely appreciate your time and support.

Part I: Demographic Information

1. Age [Please enter your age]

____

2. Gender [Select one]
○ Male
○ Female

3. Province/City of Residence [Please specify]

____

4. Type of Area [Select one]
○ Urban
○ Rural

5. Residency Duration [Select one]
○ Less than 6 months
○ 6 months to less than 1 year
○ 1 year to less than 2 years
○ 2 years to less than 5 years
○ 5 years or more

6. Highest Level of Education [Select one]
○ College or above
○ High school or equivalent
○ Below high school

7. Occupation [Select one]
○ Professional (e.g., teacher, doctor, lawyer)
○ Service industry (e.g., waiter, driver, salesperson)
○ Freelancer (e.g., writer, artist, photographer)
○ Manual worker (e.g., factory worker, construction worker, sanitation worker)

○ Office worker
○ Government/public sector employee
○ Business owner/self-employed
○ Agricultural worker (e.g., farming, forestry, fishing)
○ Other
○ Unemployed

8. Annual Household Income (CNY) [Select one]
○ Over 200,000
○ 100,000 - 200,000
○ 70,000 - 100,000
○ 40,000 - 70,000
○ Under 40,000

9. Marital and Parental Status [Select all that apply]
□ Married
□ Unmarried
□ Have children
□ No children

10. Health Insurance Type(s) [Select all that apply]
□ Urban Employee Basic Medical Insurance
□ Urban Resident Basic Medical Insurance
□ New Rural Cooperative Medical Scheme
□ Commercial Health Insurance (including “Huimin Insurance”)
□ Self-pay

Part II: Willingness and Access to Neonatal Fundus Screening

11. Before this survey, were you aware that neonatal fundus screening is recommended for newborn eye health? [Select one]
○ Yes, very aware
○ Had heard of it, but not very familiar
○ Never heard of it

12. In your opinion, how necessary is neonatal fundus screening? [Select one]
○ Very necessary
○ Somewhat necessary
○ Neutral
○ Somewhat unnecessary
○ Completely unnecessary

13. What is the maximum one-way distance you would be willing to travel for your baby to receive neonatal fundus screening? [Select one]
○ Within 10 km
○ 10 - 30 km
○ 30 - 50 km
○ Over 50 km

14a. If you had to travel to a high-level hospital for neonatal fundus screening and stay overnight, what would be your estimated out-of-pocket cost per day for accommodation?
○ No overnight stay needed (a high-level hospital is available nearby)
○ Under 100 CNY
○ 100 - 250 CNY
○ 250 - 400 CNY
○ Over 400 CNY

14b. For such a trip, what would be your estimated out-of-pocket cost per day for food?
○ No extra food needed (a high-level hospital is available nearby)
○ Under 30 CNY

○ 30 - 70 CNY
○ 70 - 150 CNY
○ Over 150 CNY

15. If a more convenient screening service (e.g., available locally) could significantly reduce travel time and cost, would you be willing to pay a slightly higher fee compared with traditional screening strategies? [Select one]
○ Very willing
○ Somewhat willing
○ Neutral
○ Somewhat unwilling
○ Completely unwilling

For the following questions (16-21), please identify hospital types based on your understanding:
Tertiary Hospitals refer to the largest and highest-level general hospitals in your region, which provide advanced medical care and typically serve as regional referral centers (e.g., Provincial People's Hospital, Municipal Central Hospital).
Secondary Hospitals refer to district-level or county-level general hospitals and maternal and child health hospitals.
Primary Care Institutions include urban community health centers and rural township hospitals or clinics.

16. How long does it take to travel from your home to a tertiary hospital? [Select one]
○ Less than 10 minutes
○ 10 - 30 minutes
○ 30 minutes - 1 hour
○ 1 - 1.5 hours
○ 1.5 - 2 hours
○ Over 2 hours (please specify) __________________

17. What type of transportation would you use to get to a tertiary hospital? [Select one]
○ Private car
○ Public transport (bus, subway)
○ Taxi or ride-hailing service
○ Walking
○ Other (please specify) __________________

18. How long does it take to travel from your home to a secondary hospital? [Select one]
○ Less than 10 minutes
○ 10 - 30 minutes
○ 30 minutes - 1 hour
○ 1 - 1.5 hours
○ 1.5 - 2 hours
○ Over 2 hours (please specify) __________________

19. What type of transportation would you use to a secondary hospital? [Select one]
○ Private car
○ Public transport (bus, subway)
○ Taxi or ride-hailing service
○ Walking
○ Other (please specify) __________________

20. How long does it take to travel from your home to a primary care institution? [Select one]
○ Less than 10 minutes
○ 10 - 30 minutes
○ 30 minutes - 1 hour
○ 1 - 1.5 hours
○ 1.5 - 2 hours
○ Over 2 hours (please specify) __________________

21. What type of transportation would you use to get to a primary care institution? [Select one]
○ Private car
○ Public transport (bus, subway)
○ Taxi or ride-hailing service
○ Walking
○ Other (please specify) ________________

22. What are the main challenges you would face in taking your baby for fundus screening? [Select all that apply]
□ Concern about transportation
□ Concern about the time commitment
□ Concern about the cost
□ Concern about potential risks of the screening
□ Lack of information on available services
□ Other (please specify) ________________

23. Do you have any questions or suggestions about neonatal fundus screening?
____

24. This survey is part of a research study. All information is collected anonymously and will be used only for scientific research. By typing "I agree" in the box below, you confirm that you understand the above, voluntarily participate in this survey, and consent to the use of your anonymized responses for research purposes.
(Please type: I agree)

S1.2 Survey Administration and Data Collection

The survey was administered between February and May 2025, at tertiary, secondary, and primary hospitals across China, covering the four major geographic regions: Eastern, Central, Western, and Northeastern. To ensure representation of regional and healthcare access disparities, both urban and rural sites were purposively selected within each region. The study targeted reproductive-aged adults (aged 25–45 years) who had resided in the sampled areas for at least two years and voluntarily agreed to participate. To ensure scientific rigor and content validity, the questionnaire was pretested in a pilot cohort of 20 participants and subsequently reviewed by an interdisciplinary expert panel comprising ophthalmologists, health policy specialists, and biostatisticians.

A total of 635 questionnaires were collected. After verification of eligibility criteria, including age, duration of residence, and informed consent, 570 responses were deemed valid for analysis, yielding an effective response rate of 89.8%. Of the valid respondents, 340 (60.0%) were from urban settings and 230 (40.0%) were from rural settings. The median age of participants was 35.0 years (interquartile range [IQR], 29.0–40.0) in urban areas and 32.0 years (IQR, 28.0–38.0) in rural areas. Female respondents accounted for 74.1% of the urban sample and 65.7% of the rural sample. The majority of respondents in both settings were married (80.0% urban vs. 83.9% rural) and had children (75.9% vs. 73.9%, respectively). Awareness of neonatal fundus screening was higher among urban respondents (75.9%) than among rural respondents (47.0%). Nevertheless, a large majority in both groups considered neonatal fundus screening to be necessary or held a neutral attitude toward its implementation (97.6% in urban areas and 92.6% in rural areas).

S1.3 Cost Derivation from Survey Data

Accommodation and food costs were estimated based on responses to Questions 14a and 14b. For each cost category, the midpoint of each predefined cost interval was multiplied by the number of respondents selecting that option, and weighted averages were calculated separately for urban and rural settings.

Transportation costs were estimated using information collected in Questions 16–21 on travel time and transportation mode, combined with a series of context-specific assumptions reflecting current travel conditions. For private car travel, fuel costs were calculated assuming a fuel consumption rate of 0.08 liters per kilometer and a fuel price of CNY 7.8 per liter. Vehicle depreciation, maintenance, and repair costs were excluded; however, a hospital parking fee of CNY 5 per visit was included. Public transportation costs (bus or subway) were estimated based on full adult fares for one accompanying parent. Taxi and ride-hailing costs were calculated using a base fare of CNY 10 for the first 2.5 kilometers, plus CNY 2 per additional kilometer and a low-speed surcharge of CNY 0.4 per minute.

Productivity losses were calculated using average annual incomes of USD 12,000 for urban residents and USD 5,160 for rural residents.

Method S2. The calculation formula of urban and rural per capita GDP

$$The\ per\ capita\ GDP\ for\ urban\ China$$

$$= \frac{China's\ per\ capita\ GDP}{(1 + \frac{1}{urban\ to\ rural\ income\ ratio}) \times urbanization\ rate}$$

$$= \frac{USD\ 13{,}445}{(1 + \frac{1}{1:0.43}) \times 67\%}$$

$$= USD\ 14033$$

$$The\ per\ capita\ GDP\ for\ rural\ China$$

$$= \frac{China's\ per\ capita\ GDP}{(1 + urban\ to\ rural\ income\ ratio) \times (1 - urbanization\ rate)}$$

$$= \frac{USD\ 13{,}445}{(1 + (1:0.43)) \times (1 - 67\%)}$$

$$= USD\ 12251$$

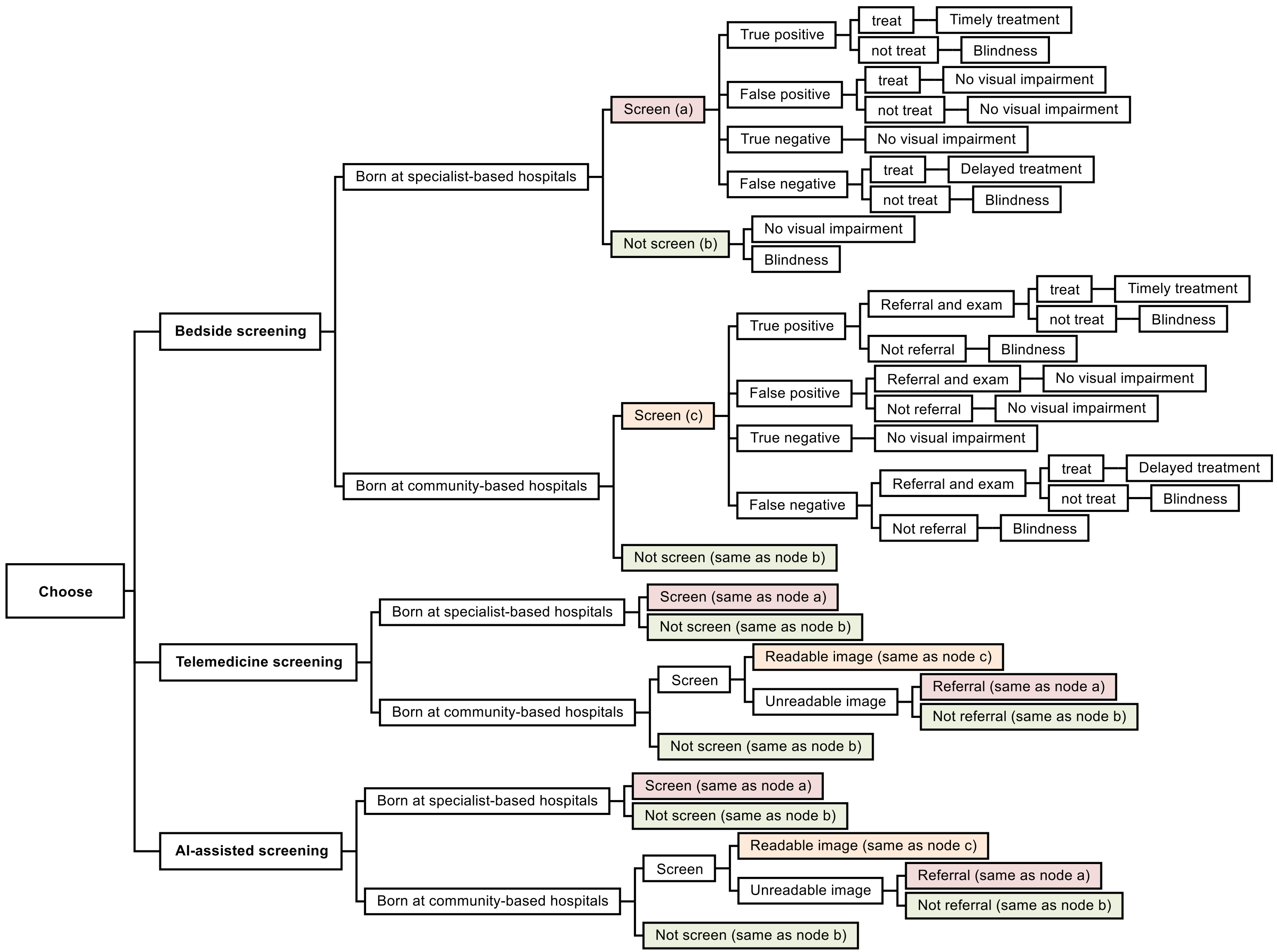

Figure S1. Decision-analytic model for retinopathy of prematurity screening strategies. The “Screen” node represents infants who receive ROP screening, either through systematic screening at healthcare facilities with screening capacity or through opportunistic case-finding at facilities without such capacity. The “Not screen” node represents infants who are not detected by either mechanism. AI – artificial intelligence.

Table S1. Variation ranges and probability distributions assumed for incidence, healthcare accessibility, diagnostic performance, utility values, and other model parameters

Variables	Urban settings					Rural settings				
	Base value	Range for test		Distribution for probabilistic sensitivity	Source	Base value	Range for test		Distribution for probabilistic sensitivity	Source
		Lower bound	Upper bound				Lower bound	Upper bound		
Incidence of TR-ROP	0.025	0.023	0.028	Beta	[1, 2]	0.025	0.023	0.028	Beta	[1, 2]
Proportion of births at...										
Specialist-based healthcare facilities	0.63	0.57	0.69	Beta	[3]	0.63	0.57	0.69	Beta	[3]
Community-based healthcare facilities	0.37	0.33	0.41	Beta	[3]	0.37	0.33	0.41	Beta	[3]
Care cascade parameters										
Screening coverage rate	0.74	0.67	0.81	Beta	[4]	0.31	0.28	0.34	Beta	[4, 5]
Opportunistic case-finding rate	0.25	0.23	0.28	Beta	[4]	0.11	0.10	0.12	Beta	[4, 5]
Referral compliance rate	0.63	0.57	0.69	Beta	[6]	0.21	0.19	0.23	Beta	[6, 7]
Treatment uptake rate	0.90	0.81	0.99	Beta	[8]	0.79	0.71	0.87	Beta	[7, 8]
Sensitivity of...										
Specialist identification of TR-ROP	0.92	0.83	1.00	Beta	[9]	0.92	0.83	1.00	Beta	[9]
Non-specialist classification of RW-ROP	0.90	0.81	0.99	Beta	[10]	0.90	0.81	0.99	Beta	[10]
AI-assisted classification of RW-ROP	1.00	0.90	1.00	Beta	[9]	1.00	0.90	1.00	Beta	[9]
Specificity of...										
Specialist identification of TR-ROP	0.99	0.89	1.00	Beta	[9]	0.99	0.89	1.00	Beta	[9]
Non-specialist classification of RW-ROP	0.87	0.78	0.96	Beta	[10]	0.87	0.78	0.96	Beta	[10]
AI-assisted classification of RW-ROP	0.98	0.88	1.00	Beta	[9]	0.98	0.88	1.00	Beta	[9]
Image readability rate	0.88	0.79	0.97	Beta	[11]	0.88	0.79	0.97	Beta	[11]
Utility for...										
No visual impairment	0.97	0.87	1.00	Beta	[12]	0.97	0.87	1.00	Beta	[12]
Timely treatment	0.70	0.63	0.77	Beta	[13]	0.70	0.63	0.77	Beta	[13]
Delayed treatment	0.52	0.47	0.57	Beta	[14, 15]	0.52	0.47	0.57	Beta	[14, 15]
Blindness	0.27	0.24	0.30	Beta	[12]	0.27	0.24	0.30	Beta	[12]
Time horizon	79	NA	NA	NA	[16]	79	NA	NA	NA	[16]
Annual discount rate	0.03	NA	NA	NA	[17]	0.03	NA	NA	NA	[17]

TR-ROP – treatment-requiring retinopathy of prematurity, RW-ROP – referral-warranted retinopathy of prematurity, AI – artificial intelligence

Table S2. Variation ranges and probability distributions assumed for screening, diagnosis, treatment, and follow-up costs

Variables	Urban settings					Rural settings				
	Base value	Range for test		Distribution for probabilistic sensitivity	Source	Base value	Range for test		Distribution for probabilistic sensitivity	Source
		Lower bound	Upper bound				Lower bound	Upper bound		
Screening (USD/person)										
Bedside screening	236	189	283	Gamma	Calculated	227	182	272	Gamma	Calculated
Telemedicine screening	281	141	422	Gamma	Calculated	268	134	402	Gamma	Calculated
AI-assisted screening	257	129	386	Gamma	Calculated	250	125	375	Gamma	Calculated
Diagnosis (USD/person)										
Referral and examination	348	278	418	Gamma	Calculated	786	629	943	Gamma	Calculated
Treatment (USD/person)										
Timely treatment	759	607	911	Gamma	Calculated	720	576	864	Gamma	Calculated
Delayed treatment	3481	2785	4177	Gamma	Calculated	3313	2650	3976	Gamma	Calculated
Blindness	8920	7136	10704	Gamma	[5]	8920	7136	10704	Gamma	[5]
Follow-up (USD/visit)										
Hospital visit follow-up	88	70	106	Gamma	Calculated	95	76	114	Gamma	Calculated
Telehealth visit follow-up	77	62	92	Gamma	Calculated	84	67	101	Gamma	Calculated
Blindness	3604	2883	4325	Gamma	[5]	3604	2883	4325	Gamma	[5]

USD – US dollars, AI – artificial intelligence

Table S3. Costs of retinopathy of prematurity screening in urban and rural China

Screening costs	Urban settings			Rural settings		
	Bedside screening	Telemedicine screening	AI-assisted screening	Bedside screening	Telemedicine screening	AI-assisted screening
Direct medical costs, USD						
Examination services	55	55	55	55	55	55
Medical equipment	0	61727	61727	0	61727	61727
Digital platforms	0	13000	29000	0	13000	29000
Medical personnel wage	24000	48000	48000	10320	20640	20640
Number of patients screened	6000	8000	15000	6000	8000	15000
Number of screening examinations	4	4	4	4	4	4
**Societal costs per person, USD**	**236**	**281**	**257**	**227**	**268**	**250**

AI – artificial intelligence, USD – US dollars

In China, fees for newborn retinal examination services range from USD 28 to USD 70 per examination, depending on local health commission standards and hospital regulations. This study applied an average examination fee of USD 55 per infant per screening episode[18].

Telemedicine and AI-assisted screening require retinal imaging equipment and digital platforms. The most widely used retinal imaging system in China is the Natus RetCam 3, with a purchase price of USD 308,634[19]. The initial setup cost for the telemedicine platform and IT support was USD 50,000, with an annual maintenance cost of USD 3,000[20]. For the AI-assisted telemedicine platform, the corresponding initial setup cost was USD 110,000, with an annual maintenance cost of USD 7,000[20]. All fixed assets were annualized over a five-year useful life, assuming no salvage value.

Under the bedside screening model, each infant required a total of 20 minutes of on-site examination performed by one ophthalmologist assisted by one nurse. In the telemedicine screening model, each screening episode required 15 minutes in total, comprising 5 minutes for fundus image acquisition by one photographer assisted by one nurse, followed by independent image interpretation by two retinal specialists at the reading center, each taking 5 minutes. Under the AI-assisted screening model, each screening episode required 8 minutes in total, including 5 minutes for fundus image acquisition by one photographer assisted by one nurse, automated initial analysis by the AI model, and subsequent independent image interpretation by two retinal specialists, each requiring 1.5 minutes.

All medical personnel were assumed to work 8 hours per day for 250 days per year. The average annual income was estimated at USD 12,000 in urban areas and USD 5,160 in rural areas; personnel costs were calculated accordingly.

Table S4. Costs of retinopathy of prematurity diagnosis in urban and rural China

Diagnosis costs	Urban settings	Rural settings
Direct medical costs, USD		
Referral services	202	674
Examination services	55	55
Medical personnel wage	4	2
Direct non-medical costs, USD		
Food and accommodation	39	34
Indirect costs, USD		
Productivity losses of parents	48	21
**Societal costs per person, USD**	**348**	**786**

USD – US dollars

Infants screened at community-based healthcare facilities may require referral to specialist-based facilities for further diagnostic evaluation. Referral service costs were estimated based on hospital ambulance fee schedules and travel distance data between facilities of different levels in urban and rural China. Diagnostic examinations used the same procedures and fee schedules as bedside screening.

Table S5. Costs of retinopathy of prematurity treatment in urban and rural China

Treatment costs	Urban settings		Rural settings	
	Timely treatment	Delayed treatment	Timely treatment	Delayed treatment
Direct medical costs, USD				
Treatment services	465	2808	465	2808
Medical personnel wage	60	90	60	90
Direct non-medical costs, USD				
Food and accommodation	90	247	112	250
Indirect costs, USD				
Productivity losses of parents	144	336	83	165
**Societal costs per person, USD**	**759**	**3481**	**720**	**3313**

USD – US dollars

According to recommendations from the Early Treatment for Retinopathy of Prematurity (ETROP) Cooperative Group[21], infants with treatment-requiring ROP received either intravitreal anti–vascular endothelial growth factor (anti-VEGF) injections or retinal laser photocoagulation within 72 hours of diagnosis. In the base-case analysis, 67% of patients were assumed to receive anti-VEGF injections at USD 555 per injection, while the remaining patients underwent laser photocoagulation at USD 281 per procedure[22, 23]. Based on these proportions, the average direct medical cost of timely treatment was estimated at USD 465 per patient. Timely treatment was performed by a multidisciplinary team of five medical professionals (one pediatric retinal specialist, two surgical assistants, one anesthesiologist, and one anesthesia assistant) and required approximately two hours to complete. Patients were assumed to be hospitalized for three days during the treatment episode.

For patients receiving delayed treatment, defined as those progressing to stage 4 or 5 ROP, vitrectomy surgery was performed at a cost of USD 2,808 per procedure[22]. This procedure was conducted by a similarly structured five-person team, required approximately three hours, and was associated with a seven-day hospitalization.

Table S6. Costs of retinopathy of prematurity follow-up care in urban and rural China

Follow-up costs	Urban settings		Rural settings	
	Hospital visit follow-up	Telehealth visit follow-up	Hospital visit follow-up	Telehealth visit follow-up
Direct medical costs, USD				
Examination services	55	55	55	55
Medical personnel wage	4	6	2	3
Direct non-medical costs, USD				
Transportation and food	5	4	28	21
Indirect costs, USD				
Productivity losses of parents	24	12	10	5
**Societal costs per person, USD**	**88**	**77**	**95**	**84**

USD – US dollars

All treated patients were assumed to fully adhere to postoperative follow-up care, with follow-up procedures similar to retinal examination services. Under the bedside screening strategy, follow-up visits were conducted at specialist-based healthcare facilities. Under the telemedicine and AI-assisted screening strategies, follow-up visits were delivered via telehealth at community-based facilities equipped with retinal imaging systems and telemedicine platforms.

Patients who received timely treatment, characterized by milder disease and a favorable prognosis, were assumed to require seven postoperative follow-up visits[24]. In contrast, patients who received delayed treatment, reflecting more severe disease and poorer prognosis, were assumed to require thirteen postoperative follow-up visits[25].

Table S7. Cost-effectiveness in scenario analyses assuming lower AI sensitivity and specificity

Strategy	Calculation method	Costs per person, USD	Incremental costs per 100,000 people, USD	Effectiveness per person, QALYs	Incremental effectiveness per 100,000 people, QALYs	ICERs, USD per QALY gained	Blindness per 100,000 people	Blindness avoided per 100,000 people
Scenario analysis (71% AI sensitivity and 74% AI specificity)								
**Urban settings**								
AI screening	-	1522	-	29.7086	-	-	1063	-
Telescreening	AI vs tele	1514	807341	29.7126	-400	-2018	1063	0
Bedside screening	AI vs bedside	1758	-23605344	29.6794	2925	-8070	1320	257
	Tele vs bedside	1758	-24412685	29.6794	3326	-7340	1320	257
**Rural settings**								
AI screening	-	2585	-	29.5828	-	-	2067	-
Telescreening	AI vs tele	2583	210622	29.5833	-49	-4298	2067	0
Bedside screening	AI vs bedside	2596	-1090742	29.5794	348	-3134	2097	30
	Tele vs bedside	2596	-1301364	29.5794	397	-3278	2097	30

USD – US dollars, QALY – quality-adjusted life year, ICER – incremental cost-effectiveness ratio, AI – artificial intelligence

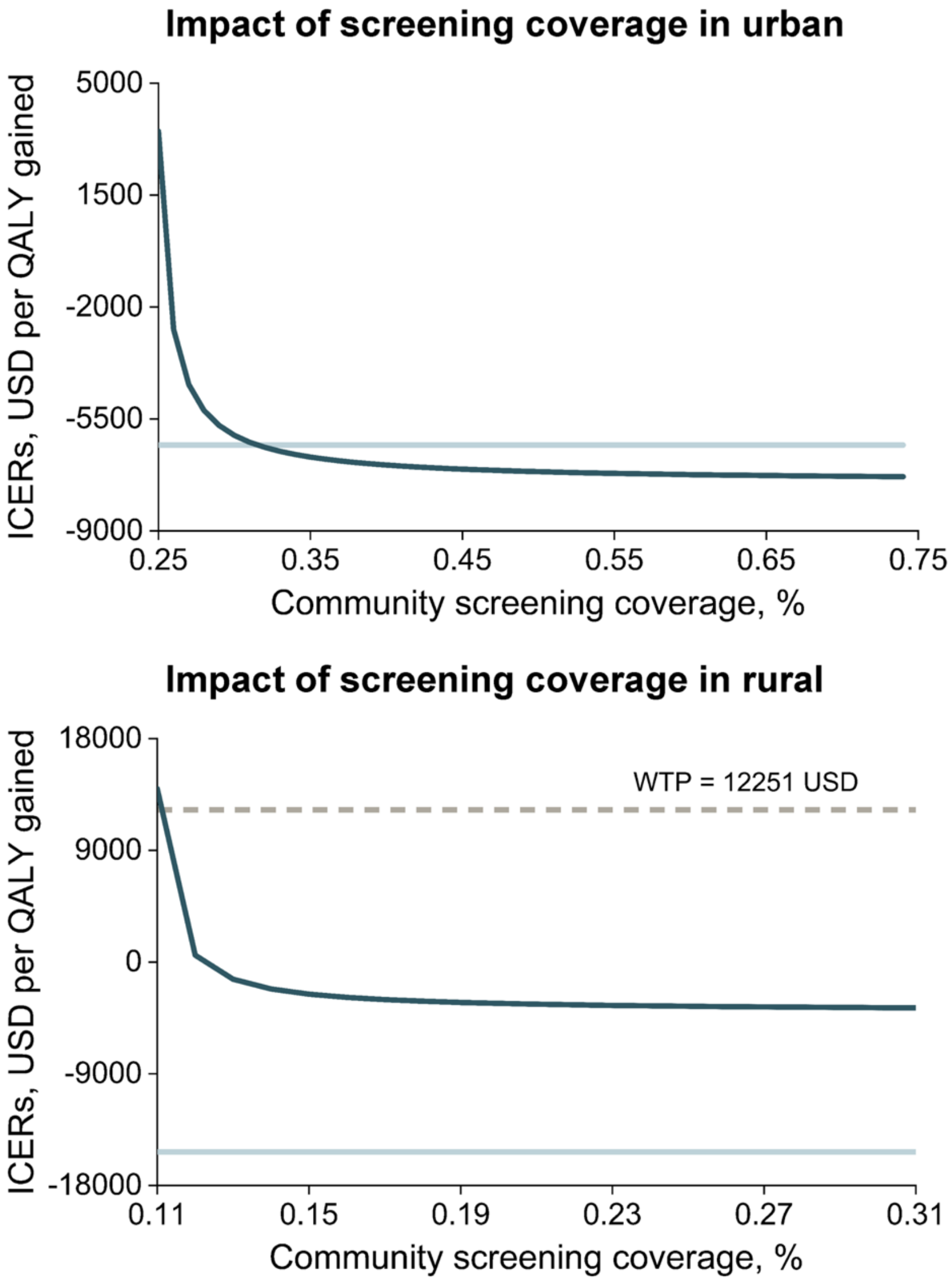

Figure S2. Scenario analysis of screening coverage in community-based healthcare facilities in urban and rural settings. Dark blue lines represent AI-assisted screening compared with bedside screening, while light blue lines represent AI-assisted screening compared with telemedicine screening. ICER – incremental cost-effectiveness ratio, USD – US dollars, QALY – quality-adjusted life year, WTP – willingness-to-pay.

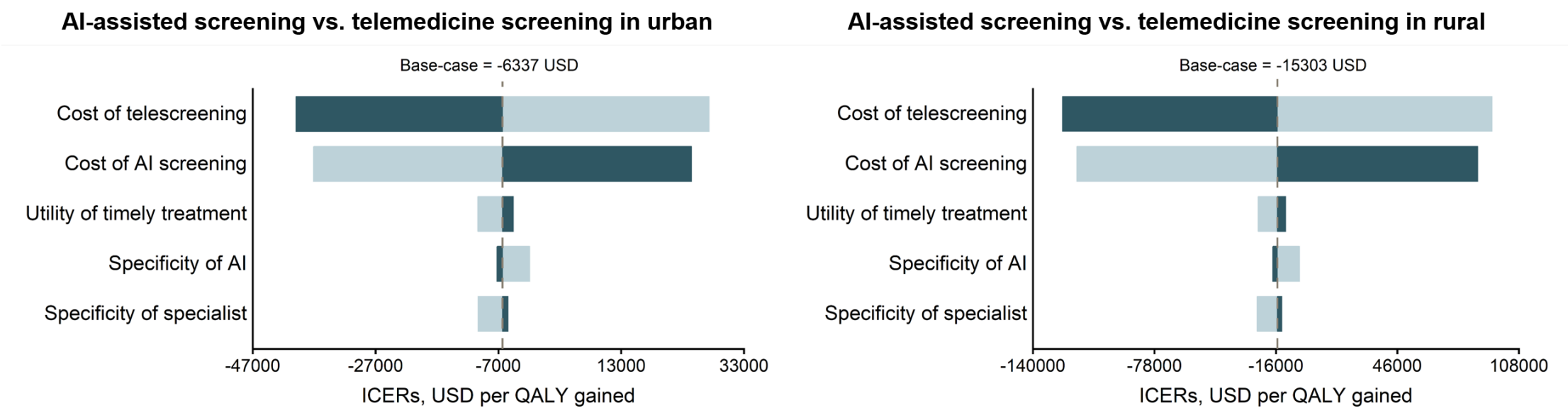

Figure S3. Tornado plot of one-way sensitivity analyses comparing AI-assisted screening with telemedicine screening in urban and rural settings. Dark blue bars represent ICERs when parameter values are set to their upper bounds, while light blue bars represent ICERs when parameter values are set to their lower bounds. AI – artificial intelligence, USD – US dollars, ICER – incremental cost-effectiveness ratio, QALY – quality-adjusted life year.

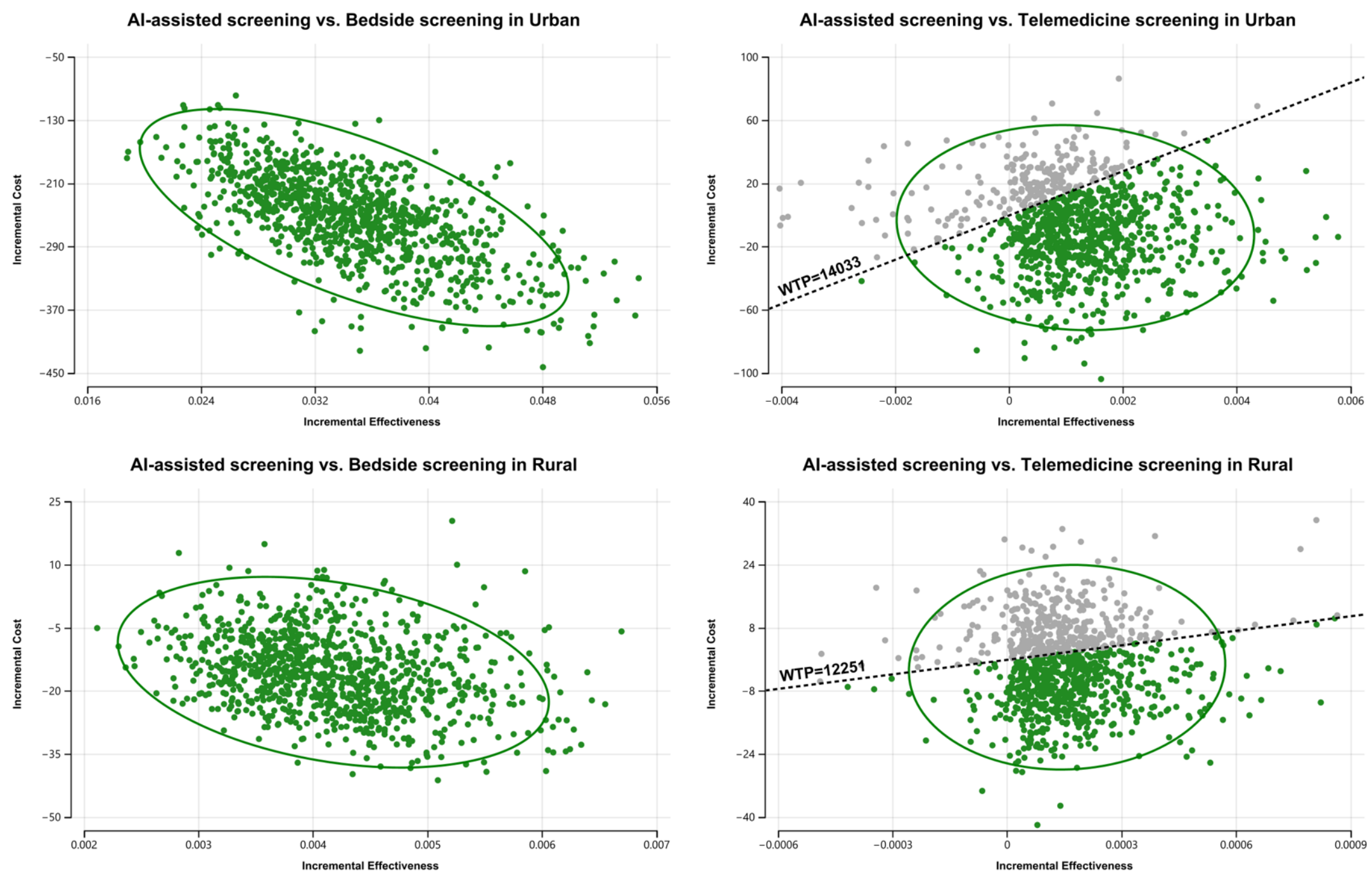

Figure S4. Scatterplot of probabilistic sensitivity analyses comparing AI-assisted screening with bedside screening and telemedicine screening in urban and rural settings. Green points indicate cost-effective iterations, while grey points indicate non–cost-effective iterations. The dashed line indicates the WTP threshold, while the ellipse denotes the 95% confidence interval. Across 1,000 Monte Carlo simulations, AI screening was cost-effective versus bedside screening in 100% of iterations in both settings, and versus telemedicine in 75.7% (urban) and 59.2% (rural) of iterations. AI – artificial intelligence, WTP – willingness-to-pay.

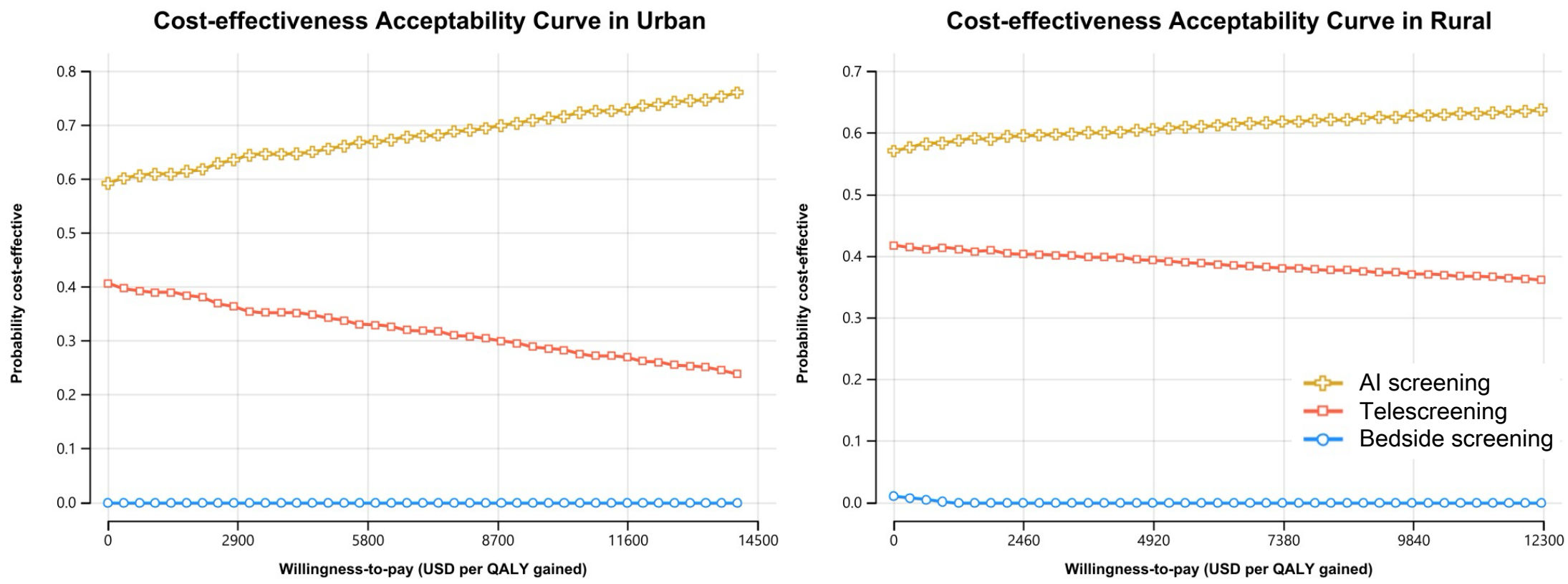

Figure S5. Cost-effectiveness acceptability curves comparing three screening strategies in urban and rural settings. The curves show the probability that each strategy is cost-effective over a range of willingness-to-pay thresholds. AI screening was the most likely strategy to be cost-effective as willingness-to-pay changes, with the probability ranging from 59.3% to 76.1% in urban and 57.1% to 63.8% in rural. AI – artificial intelligence, USD – US dollars, QALY – quality-adjusted life year.

Table S8. Consolidated Health Economic Evaluation Reporting Standards checklist

Topic	No.	Item	Location where item is reported
Title			
	1	Identify the study as an economic evaluation and specify the interventions being compared.	Page 1
Abstract			
	2	Provide a structured summary that highlights context, key methods, results, and alternative analyses.	Page 1
Introduction			
Background and objectives	3	Give the context for the study, the study question, and its practical relevance for decision making in policy or practice.	Page 2
Methods			
Health economic analysis plan	4	Indicate whether a health economic analysis plan was developed and where available.	Not reported
Study population	5	Describe characteristics of the study population (such as age range, demographics, socioeconomic, or clinical characteristics).	Page 3
Setting and location	6	Provide relevant contextual information that may influence findings.	Page 3-4
Comparators	7	Describe the interventions or strategies being compared and why chosen.	Page 3-4
Perspective	8	State the perspective(s) adopted by the study and why chosen.	Page 5
Time horizon	9	State the time horizon for the study and why appropriate.	Page 4
Discount rate	10	Report the discount rate(s) and reason chosen.	Page 4-5
Selection of outcomes	11	Describe what outcomes were used as the measure(s) of benefit(s) and harm(s).	Page 3-5
Measurement of outcomes	12	Describe how outcomes used to capture benefit(s) and harm(s) were measured.	Page 4-5
Valuation of outcomes	13	Describe the population and methods used to measure and value outcomes.	Page 3-5
Measurement and valuation of resources and costs	14	Describe how costs were valued.	Page 5
Currency, price date, and conversion	15	Report the dates of the estimated resource quantities and unit costs, plus the currency and year of conversion.	Page 5
Rationale and description of model	16	If modeling is used, describe in detail and why used. Report if the model is publicly available and where it can be accessed.	Page 2-4
Analytics and assumptions	17	Describe any methods for analyzing or statistically transforming data, any extrapolation methods, and approaches for validating any model used.	Page 4-5
Characterizing heterogeneity	18	Describe any methods used for estimating how the results of the study vary for subgroups.	Page 5-6
Characterizing distributional effects	19	Describe how impacts are distributed across different individuals or adjustments made to reflect priority populations.	Page 6
Characterizing uncertainty	20	Describe methods to characterize any sources of uncertainty in the analysis.	Page 5-6
Approach to engagement with patients and others affected by the study	21	Describe any approaches to engage patients or service recipients, the general public, communities, or stakeholders (such as clinicians or payers) in the design of the study.	Page 3-5

Results			
Study parameters	22	Report all analytic inputs (such as values, ranges, references) including uncertainty or distributional assumptions.	Page 3-5
Summary of main results	23	Report the mean values for the main categories of costs and outcomes of interest and summarize them in the most appropriate overall measure.	Page 6
Effect of uncertainty	24	Describe how uncertainty about analytic judgments, inputs, or projections affect findings. Report the effect of choice of discount rate and time horizon, if applicable.	Page 7-8
Effect of engagement with patients and others affected by the study	25	Report on any difference patient/service recipient, general public, community, or stakeholder involvement made to the approach or findings of the study.	Not reported
Discussion			
Study findings, limitations, generalizability, and current knowledge	26	Report key findings, limitations, ethical or equity considerations not captured, and how these could affect patients, policy, or practice.	Page 8-9
Other relevant information			
Source of funding	27	Describe how the study was funded and any role of the funder in the identification, design, conduct, and reporting of the analysis	Page 10
Conflicts of interest	28	Report authors conflicts of interest according to journal or International Committee of Medical Journal Editors requirements.	Page 10

References

1. Fei P, Liu Z, He L, Li N, Xu L, Zhang M, et al. Early detection of ocular abnormalities in a Chinese multicentre neonatal eye screening programme-1-year result. Acta Ophthalmol. 2021;99:e415–22.
2. Zhong Y, Lin X, Yang Y, He L, Li N, Lu J, et al. Screening for retinopathy of prematurity in China: a five-year cohort study in seven screening centers. BMC Ophthalmol. 2025;25:3.
3. Li Q, Li X, Zhang Q, Zhang Y, Liu L, Cheng X, et al. A Cross-Sectional Nationwide Study on Accessibility and Availability of Neonatal care Resources in Hospitals of China: Current Situation, Mortality and Regional Differences: Neonatal Care Resources and Newborn Mortality in China. Lancet Reg Health West Pac. 2021;14:100212.
4. Wang H, Luo R. Status of the occurrence and screening of retinopathy of prematurity. Chin J Women Child Health. 2022;13:1–5.
5. Li R, Yang Z, Zhang Y, Bai W, Du Y, Sun R, et al. Cost-effectiveness and cost-utility of traditional and telemedicine combined population-based age-related macular degeneration and diabetic retinopathy screening in rural and urban China. Lancet Reg Health West Pac. 2022;23:100435.
6. Wang X, Zhao G, Ma X, Zhang H, Zhang J, Yang L, et al. Effect of multidisciplinary health education in screening of retinopathy in premature infants. J Clin Med Pract. 2020;24:123–5.
7. Tang J, Liang Y, O'Neill C, Kee F, Jiang J, Congdon N. Cost-effectiveness and cost-utility of population-based glaucoma screening in China: a decision-analytic Markov model. Lancet Glob Health. 2019;7:e968–78.
8. Zhu Y, Jiang S, Wang X, Li M, Yang H, Cao Y, et al. Incidence of retinopathy of prematurity among infants less than 34 weeks' gestation before discharge in 25 NICUs in China: A cross-sectional survey. Chin J Evid Based Pediatr. 2020;15:45–9.
9. Wang J, Ji J, Zhang M, Lin JW, Zhang G, Gong W, et al. Automated Explainable Multidimensional Deep Learning Platform of Retinal Images for Retinopathy of Prematurity Screening. JAMA Netw Open. 2021;4:e218758.
10. Quinn GE, Ying GS, Daniel E, Hildebrand PL, Ells A, Baumritter A, et al. Validity of a telemedicine system for the evaluation of acute-phase retinopathy of prematurity. JAMA Ophthalmol. 2014;132:1178–84.
11. Li L, Lin D, Lin Z, Li M, Lian Z, Zhao L, et al. DeepQuality improves infant retinopathy screening. NPJ Digit Med. 2023;6:192.
12. Quinn GE, Dobson V, Saigal S, Phelps DL, Hardy RJ, Tung B, et al. Health-related quality of life at age 10 years in very low-birth-weight children with and without threshold retinopathy of prematurity. Arch Ophthalmol. 2004;122:1659–66.
13. Jackson KM, Scott KE, Graff Zivin J, Bateman DA, Flynn JT, Keenan JD, et al. Cost-utility analysis of telemedicine and ophthalmoscopy for retinopathy of prematurity management. Arch Ophthalmol. 2008;126:493–9.
14. Karacorlu M, Hocaoglu M, Sayman Muslubas I, Arf S. Long-term functional results following vitrectomy for advanced retinopathy of prematurity. Br J Ophthalmol. 2017;101:730–4.
15. Brown GC. Vision and quality-of-life. Trans Am Ophthalmol Soc. 1999;97:473–511.
16. Xinhua News Agency. Chinese per capita life expectancy in 2024 increased by 1.1 years compared to 2020. Central People's Government of the People's Republic of China; 11 September 2025. Available: https://www.gov.cn/lianbo/bumen/202509/content_7040166.htm. Accessed: 09 January 2026.
17. Sanders GD, Neumann PJ, Basu A, Brock DW, Feeny D, Krahn M, et al. Recommendations for Conduct, Methodological Practices, and Reporting of Cost-effectiveness Analyses: Second Panel on

Cost-Effectiveness in Health and Medicine. JAMA. 2016;316:1093–103.
18. Peking University Third Hospital. Diagnosis and Treatment Team for Retinopathy of Prematurity. Beijing, China: Peking University Third Hospital; 02 April 2025. Available: https://puh3.net.cn/info/2901/150141.htm. Accessed: 09 January 2026.
19. Fudan University Children's Hospital. Announcement of Winning Bid for Pediatric Hospital Ophthalmology Wide-Field Imaging System Procurement Project. Shanghai, China: Fudan University Children's Hospital; 22 November 2024. Available: https://ch.shmu.edu.cn/uploads/file/20241126/1732581503910229.pdf. Accessed: 09 January 2026.
20. Liu H, Li R, Zhang Y, Zhang K, Yusufu M, Liu Y, et al. Economic evaluation of combined population-based screening for multiple blindness-causing eye diseases in China: a cost-effectiveness analysis. Lancet Glob Health. 2023;11:e456–65.
21. Early Treatment for Retinopathy of Prematurity Cooperative Group. Revised indications for the treatment of retinopathy of prematurity: results of the early treatment for retinopathy of prematurity randomized trial. Arch Ophthalmol. 2003;121:1684–94.
22. Fan J, Zhang M, Zhao W, Han Y, Hu W, Jia H, et al. Health economic evaluation of telemedicine diabetic retinopathy examination using a non-mydriatic fundus camera in China. Chin J Ophthalmol. 2023;59:919–29.
23. Le TP, Feng J, Ding L, Hu R, Lou XB, Ulrich JN, et al. Survey of current retinopathy of prematurity practices in China. Int J Ophthalmol. 2021;14:1241–7.
24. Zhang G, Yang M, Zeng J, Vakros G, Su K, Chen M, et al. Comparison of Intravitreal Injection of Ranibizumab Versus Laser Therapy for Zone II Treatment-Requiring Retinopathy of Prematurity. Retina. 2017;37:710–7.
25. Li X, Cao X, Li X, Yin H, Liang J. Therapeutic effect of segmental scleral buckling and vitreoretinal surgery for stage 4 and 5 retinopathy of prematurity. Chin J Ocul Fundus Dis. 2016;32:505–9.
